# Highly Selective Tilted Triangular Springs with Constant Force Reaction †

**DOI:** 10.3390/s24051677

**Published:** 2024-03-05

**Authors:** Lisa Schmitt, Philip Schmitt, Martin Hoffmann

**Affiliations:** Microsystems Technology, Faculty of Electrical Engineering and Information Technology, Ruhr University Bochum, 44801 Bochum, Germany; philip.schmitt@rub.de (P.S.); martin.hoffmann-mst@rub.de (M.H.)

**Keywords:** micromechanical spring, constant force mechanism, MEMS, electrostatic actuator

## Abstract

Guiding mechanisms are among the most elementary components of MEMS. Usually, a spring is required to be compliant in only one direction and stiff in all other directions. We introduce triangular springs with a preset tilting angle. The tilting angle lowers the reaction force and implements a constant reaction force. We show the influence of the tilting angle on the reaction force, on the spring stiffness and spring selectivity. Furthermore, we investigate the influence of the different spring geometry parameters on the spring reaction force. We experimentally show tilted triangular springs exhibiting constant force reactions in a large deflection range and a comb-drive actuator guided by tilted triangular springs.

## 1. Introduction

Guiding mechanisms enable translational in-plane displacement of Micro-Electro-Mechanical Systems (MEMS) and are, therefore, among the most important components of microsystems technology. In MEMS, linear guiding mechanisms are used in a wide variety of applications such as in electrostatic actuators [1], or in inertial MEMS to guide proof masses. Usually, these applications require a guiding mechanism that is compliant in the displacement direction and stiff in all other directions. For a solid spring, this means that it should have a low stiffness, *k_x_*, in the deflection direction, *x* (i.e., the slope of the force–displacement characteristic (FDC)), but high stiffnesses in the orthogonal *y*-direction (*k_y_*). The ratio of the stiffness can be defined as the selectivity, *S*, with

(1) $${\left.S=\frac{k_{y}}{k_{x}}\right|}_{y\to 0}$$

**Figure 1:**
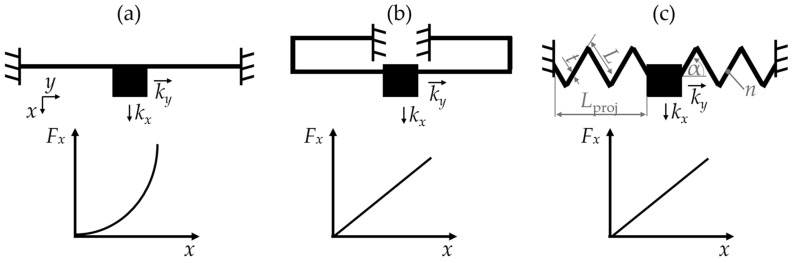
A sketch of the FDC of a (**a**) clamped–clamped beam, (**b**) serpentine spring, (**c**) triangular spring [2,3].

Guiding mechanisms have many different forms of appearance and geometries. We give a comprehensive overview of the guiding mechanisms in [2]. The clamped–clamped beams are among the most common guiding mechanisms [1,3] that fit the requirements for guiding springs quite well, as their stiffness in the displacement direction, *k_x_*, is much smaller than their stiffness in the direction orthogonal to the displacement, *k_y_*. Figure 1a shows a clamped–clamped beam and its typical non-linear FDC [4]. Consequently, the mechanical reaction force, *F_x_*, of the clamped beams increases strongly with progressing displacement. This characteristic is quite unattractive for, e.g., electrostatic actuators. Serpentine springs have a linear FDC, as shown in Figure 1b; hence the mechanical force, *F_x_*, increases only slightly during deflection, making the serpentine springs attractive for electrostatic comb-drive actuation [1,5]. However, serpentine springs only realize a uni-directional displacement and suffer from a strongly decreasing selectivity with progressing displacement that results, e.g., in the side instability of comb-drive actuators [1].

Springs with an M-shaped geometry have a non-linear and asymmetric FDC [6]. Both triangular and sinusoidal springs, presented in [2], have a geometry similar to the M-shaped springs. The triangular springs (Figure 1c) are characterized by a linear FDC, high selectivity, and a large displacement range. They achieve large deflections without rupture and can be deflected in both the positive and negative *x*-directions. The appearance of these springs is highly variable, since the number of spring segments *n*, the angle of inclination α as well as the thickness, *t*, and the length, *L*, can be varied. The triangular springs can be migrated into sinusoidal shaped springs, which reduces the stress peaks that occur in the spring kinks and limits the maximum deflection of the spring [2].

There are also springs with a constant FDC [7]. The springs realize a constant force feedback when applying an external force, e.g., a voltage [8,9]. Consequently, the resulting force is not proportional to the deflection [9]. In the constant force range, the stiffness, *k_x_*, of such springs is ideally 0 N/m. Constant-force springs are either used to maintain the functionality of sensitive MEMS, which could be affected by fluctuations of the spring force [10] or for specific applications that require a constant force [11]. A constant force can be accomplished by complex force feedback systems that control the force by means of an actuator [12,13]. To generate constant forces in microsystems, electrostatic actuators with closed-loop feedback control are often used to control the force in MEMS [14]. Alternatively, the spring itself can be used to generate a constant and displacement-independent force. Such systems include, for example, buckling beams combined with a linear spring [8,11,15]. Here, the FDC of the negative buckling spring is superimposed with the FDC of the linear spring. The superposition of both springs results in a displacement independent constant force reaction [11,16,17,18,19].

In this contribution, we present a tilted triangular shaped spring achieving constant force reactions. Unlike other solutions, the tilted triangular spring does not require a superimposing of multiple individual reaction forces making it a simple and compact solution for a linear guidance with a constant force. In Section 2, we present the spring design and discuss the simulation results. Here, we take a close look at the influence of the design parameters on the constant force range. In Section 3, we present the experimental setup and discuss the results obtained by the experiments. In Section 4, we show a comb-drive actuator displaced by a tilted triangular spring. Section 5 gives a short summary of this article.

## 2. Materials and Methods

### 2.1. Spring Design

**Figure 2:**
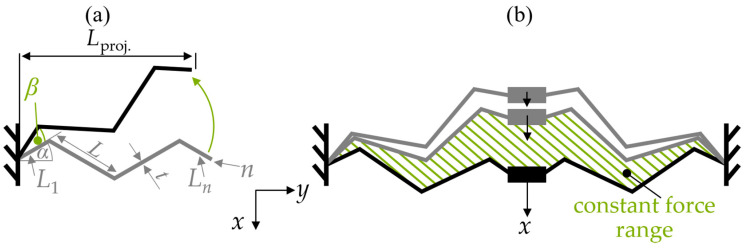
(**a**) A sketch of the tilted triangular spring with a preset tilting angle, *β*, (**b**) displacement of the tilted triangular spring with a constant force region.

The tilted triangular spring is characterized by a tilting angle, *β*, as shown in Figure 2a. The properties of this spring are defined by the number of straight elements, *n*, their length, *L*, the thickness, *t*, depth, *d*, of the device layer, and the inclination angle, *α*. Figure 2 also shows the projected length, *L*_proj_. Based on the results that we achieved in [2], we aim to achieve symmetrical force-displacement curves. Therefore, we only used springs with an even number of beams, i.e., *n* = 2, 4, 6, …, and the length of the first, *L*_1_, and the last beam, *L_n_*, is half the length of the other beams, i.e., *L*/2. The triangular spring achieves a constant force reaction with a preset initial tilting angle, *β*, as an additional degree of freedom. To realize a translational displacement, we use a symmetric design (Figure 2b). As shown in Figure 2b, the constant force range (CFR) arises with the progressing displacement of the spring; consequently, the tilted triangular spring achieves a partially constant spring force.

### 2.2. Simulation Procedure

**Figure 3:**
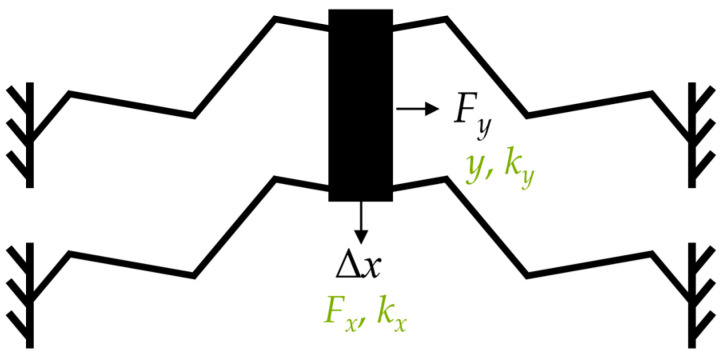
Simulation setup; applied force or displacement (black); resulting values (green).

The springs are simulated by FEM using COMSOL *Multiphysics*. We simulate a system with four identical springs with identical design parameters (Figure 3) to guarantee a translational displacement and to maintain the system stability. The structures are simulated with respect to the geometric non-linearity.

The springs are displaced in defined steps, ∆x, resulting in a change in the mechanical reaction force of the spring, ${∆F}_{x}$. With

(2) $$k_{x}=\frac{∆F_{x}}{∆x}$$

we determine the stiffness, $k_{x}$, in the displacement direction. In a second simulation, a constant force, $F_{y}$, also acts in the *y*-direction, resulting in a displacement, *y*. With

(3) $$k_{y}=\frac{F_{y}}{y}$$

we determine the stiffness, $k_{y}$, of the spring when displaced in the *x*-direction. The selectivity, *S*, of the spring is given by (1) as the quotient of $k_{y}$ and $k_{x}$.

**Figure 4:**
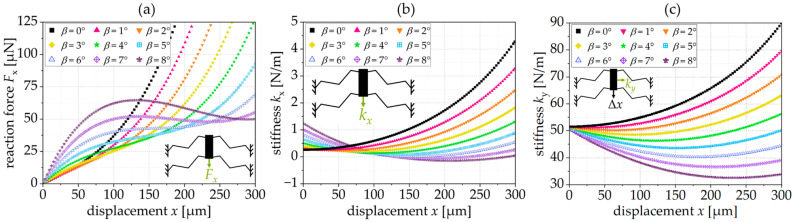
(**a**) Reaction force, $F_{x}$, as a function of *β*, (**b**) stiffness, $k_{x}$, as a function of *β*, (**c**) stiffness, $k_{y}$, as a function of *β*; simulation results for an exemplary spring with *L* = 400 µm, *t* = 5 µm, *n* = 6 and *α* = 20°.

**Figure 5:**
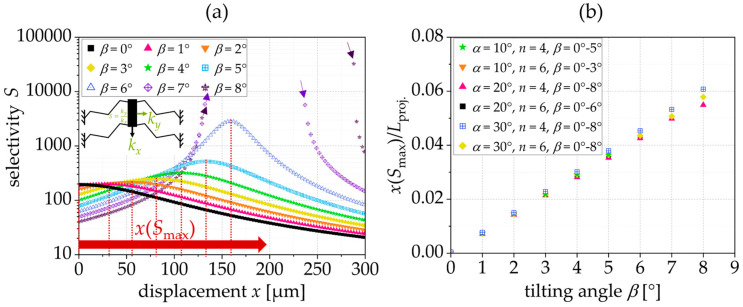
(**a**) Selectivity, *S*, as a function of *β*; (**b**) position of maximum selectivity, *x*(*S*_max_), normalized to *L*_proj._ depending on the tilting angle, *β*; simulation results for an exemplary spring with *L* = 400 µm and *t* = 5 µm.

**Figure 6:**
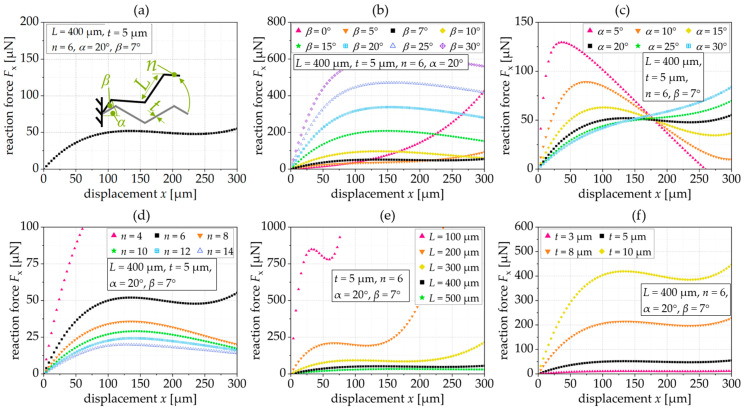
Simulated force–displacement characteristic of (**a**) a standard spring (*L* = 400 µm, *t* = 5 µm, *n* = 6, *β=7°* and *α* = 20°), when varying (**b**) the tilting angle, *β*, (**c**) the inclination angle, *α*, (**d**) the number of beams, *n*, (**e**) the beam length, *L*, (**f**) the beam thickness, *t*.

The design parameters are given in Table 1. To analyze the influence of the tilting angle on the reaction force, stiffness, and selectivity, we designed triangular springs with the ability to vary the tilting angle, *β*, from 0° to 8° as shown in Figure 4 and Figure 5. To analyze the correlation between the position of the maximum selectivity, *x*(*S*_max_), the projected length, *L*_proj._, and the tilting angle, the tilting and inclination angle, as well as the number of beams were varied in Figure 5b. In Figure 6, the influence of the design parameters was investigated. Therefore, in each diagram, one single parameter was varied and the influence on the constant force range was analyzed.

### 2.3. Simulation Results

#### 2.3.1. Constant Force Range (CFR) and Selectivity

Figure 4a shows the simulated force reaction of a triangular spring for different preset tilting angles *β*. For tilting angles from 0° to 5°, the reaction force increases continuously. For tilting angles of 6° and 7°, the spring exhibits an approximately constant force range. For the selected geometry, the force reaction shows local maxima and minima for a tilting angle *β* = 8° or higher.

Figure 4b shows the resulting spring stiffness, $k_{x}$, in the displacement direction, *x*. For the springs with constant force reaction (*β* = 6° and 7°), the stiffness is approx. 0 N/m in the CFR. As shown in Figure 4c, the stiffness orthogonal to the displacement, $k_{y}$, slightly increases for springs with no or small tilting angles, whereas springs with larger tilting angles have a slightly decreasing stiffness, $k_{y}$.

Figure 5a shows the resulting selectivity, *S*. Apart from generating a constant force, tilted triangular springs exhibit a high level of selectivity, as defined by the stiffness ratio between the *y*- and *x*-directions (cf. (1)). With the increase in the tilting angle, the maximum selectivity, *S*_max_, increases, too. Consequently, the tilted triangular spring exhibits a selectivity, *S*, as a function of the tilting angle. Thereby, the position of the maximum selectivity, *x*(*S*_max_), shifts towards larger displacements. Springs with a reaction force featuring a local maximum and minimum do not have a defined maximum of selectivity. The increase in selectivity is important, e.g., for the lateral instability of the comb-drive actuators. A higher selectivity allows a higher voltage, $U_{SI}$, to be applied, and thus allows larger displacements before lateral-side instability occurs. This correlation results from the analytical model for the displacement of comb-drive actuators that is highly dependent on the selectivity, *S*, [1].

(4) $$U_{SI}^{2}=\frac{b^{2}k_{x}}{2\varepsilon_{0}\varepsilon_{r}dn_{e}}\left(\sqrt{2\frac{k_{y}}{k_{x}}+\frac{x_{0}^{2}}{b^{2}}}-\frac{x_{0}}{b}\right)=\frac{b^{2}k_{x}}{2\varepsilon_{0}\varepsilon_{r}tn_{e}}\left(\sqrt{2S+\frac{x_{0}^{2}}{b^{2}}}-\frac{x_{0}}{b}\right)$$

Here, $U_{SI}$ is the voltage when side instability occurs, *b* is the distance between two electrodes, *n_e_* is the number of electrodes, *d* is the thickness of the device layer, and *x*_0_ is the initial overlap of electrodes.

Figure 5b shows that the position of maximum selectivity normalized to the projected spring length, *L*_proj._, depends only on the tilting angle, *β*, and increases almost linearly with it. Consequently, a spring with a tilting angle of, e.g., *β* = 6° reaches its maximum selectivity at a deflection of about 4.5% of its projected length.

#### 2.3.2. Influence of the Spring Parameter on the Constant Force Range

To better analyze the influence of the different geometry parameters of the tilted triangular spring on the constant force reaction, we use a standard spring with a force reaction, as shown in Figure 6a, with a constant force of 50.1 ± 1.5 µN in the range of 100 µm to 250 µm. In Figure 6b–f, we vary a single geometry parameter.

Figure 6b shows the influence of the tilting angle, *β*, on the constant force. At small tilting angles (in this case, *β* = 5°), no constant force range is achieved, while at larger tilting angles, the force response decreases slightly (negative stiffness) after reaching a maximum.

Figure 6c shows the influence of the inclination angle, *α*, which in this case defines the constant force range at 20°. If the inclination angle decreases, the reaction force increases sharply before it drops sharply. For a larger *α* (here, *α =* 30°), the reaction force increases with increasing deflection without establishing an extended constant force range.

Figure 6d shows the influence of the number of beam segments, *n*. If the spring consists of few beam segments, in this case *n* = 4, there is no constant force; if there are many beam segments, the reaction force decreases after reaching a maximum.

Figure 6e shows that springs with long segments have a low reaction force and a large linear force range. If the segments of the tilted triangular spring are short, in this case 100 µm and 200 µm, respectively, the spring force is higher and the constant force range is non-existent or very small.

The segment thickness, *t*, is used to set the level of the reaction force, so that as the thickness decreases, the reaction force decreases and the constant force range increases, as shown in Figure 6f.

The optimal design depends on the application and the design of interacting components on the chip. The design can be optimized to, e.g., a large constant force range, a very high or very low constant force, or a spring with a small length or height. An example of this is as follows: (1) If a large constant force range is required, it is useful to have a long beam length, *L*, or thin beam thickness, *t*. However, large beams require a lot of space, and the beam thickness can be limited by the quality of the etching process or the thickness of the device layer. (2) If a constant and high reaction force is required, the tilting angle or the thickness can be increased. However, a spring with a high tilting angle requires lots of space. Consequently, the optimal design always depends on the application, other chips’ design properties or the etching processes, and we cannot derive a single expression for an optimal spring design.

### 2.4. Fabrication

Based on the simulations conducted, demonstrator springs were designed. In addition to that, an electrostatic comb-drive actuator was designed to provide the tilted springs with a constant force.

**Figure 7:**
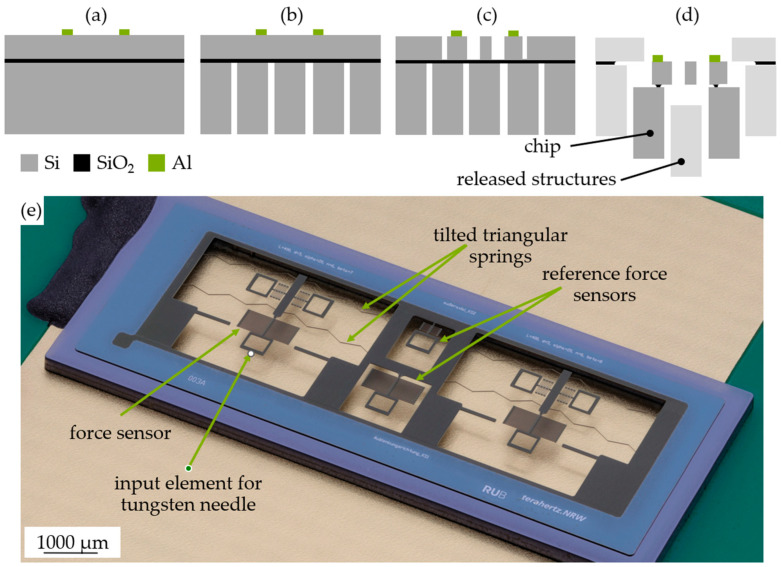
(**a**–**d**) Fabrication flowchart, (**e**) stacking photo of the fabricated chip.

The chips were manufactured in a dicing-free process for silicon-on-insulator (SOI) substrates known from [20] using a device layer of 20 µm and a handle layer of 300 µm; this is shown in Figure 7. First, a 100 nm aluminum layer was sputtered on the device layer. The aluminum was used for the electrostatic activation of the comb-drive electrodes. Afterwards, the electrical contacts were patterned by lithography and wet chemical etching, so that the mechanical structures were not covered with aluminum (Figure 7a). In the next step, we etched the handle layer by deep reactive ion etching (DRIE) using a SiO_2_-hard mask (Figure 7b). To structure the device layer, we used a hard mask of aluminum nitride (AlN) and DRIE (Figure 7c). The chips were released from the substrate by hydrofluoric vapor etching (Figure 7d). Afterwards, the chips were fixed on PCBs. The simulated thickness of the springs was 5 µm; however, the fabricated thickness was 4.8 µm. Figure 7e shows a stacking photo of a fabricated chip with the spring demonstrator. Each spring system consists of a slider with two tilted guiding springs on each side. We provided the springs with additional force sensors to experimentally determine the force reaction on-chip. The chips also contain reference force sensors to determine the stiffness of the force sensors.

### 2.5. Experimental Charcterization Procedure

**Figure 8:**
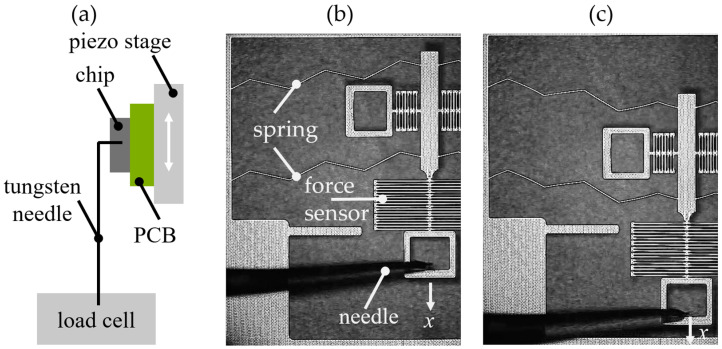
(**a**) sketch of the setup for calibrating the force sensor; left-hand-side of the tilted triangular spring in (**b**) the initial position, and (**c**) the displaced spring.

First, the integrated force sensors were calibrated. Therefore, we could determine the spring constant, *k*_FS_, of the force sensor. Instead of using the springs that were already connected to the tested device, we used the reference force sensors shown in Figure 7e. The calibration procedure included the simultaneous measurement of force and displacement of the force sensor, as illustrated in Figure 8a. The chips were mounted on a piezoelectric stage (PI Q-545). A tungsten needle was inserted into the input element of the reference force sensor. This needle was connected to a load cell (Sartorius WZA224-ND) with a resolution of 0.1 μN. The piezo stage was shifted upwards stepwise in defined increments. This resulted in an expansion of the force sensor and changed the force reaction measured by the load cell. Using (2), the spring constants of the reference force sensors were measured to be 0.2 N/m for force sensor 1 and 0.55 N/m, 1.5 N/m, and 4.6 N/m for force sensors 2, 3, and 4, respectively.

Knowing the spring constant of the force sensor, the triangular springs could be characterized using the integrated force sensors on the device under test. Therefore, again, a needle was connected to the input element of the force sensor and a displacement controlled by a piezo stage was imposed at the spring, as shown in Figure 8b. Both the deflection of the slider and the extension of the force sensor were measured visually with a microscope camera. With

(5) $$F_{DUT}(x)=F_{FS}(x)=k_{FS}x$$

the force was measured as a function of the spring displacement, which yielded the FDC. A deflected spring is shown in Figure 8c. Each experiment was repeated three times and the mean values and a standard deviation were calculated.

## 3. Experimental Results and Discussion

### 3.1. Overview of the Results

Table 2 summarizes the parameters of the selected spring designs.

### 3.2. Characterization of the Tilted Triangular Springs

We designed triangular springs where the tilting angle varied from 0° to 8°. The design parameters of the spring are given in Table 2.

**Figure 9:**
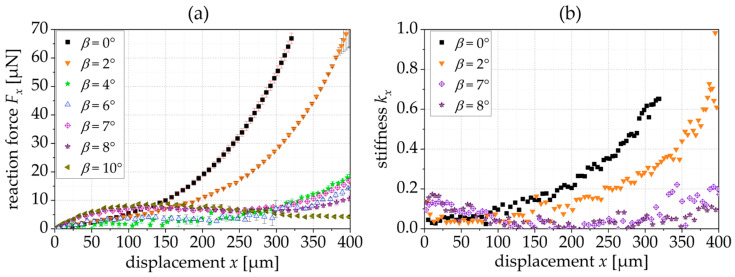
(**a**) Experimentally derived FDC of triangular springs when varying the tilting angle *β*; (**b**) resulting stiffness $k_{x}$ (*L* = 400 µm, *t* = 4.8 µm, *n* = 6 and *α* = 20°).

Figure 9a shows the experimentally derived reaction force depending on the tilting angle *β*. As shown in the simulation results, a constant force appears within the span of *β* = 6° to *β* = 8°. For *β* = 10°, and the force reaction curve has a local maximum. The experimentally derived reaction force (6.8 ± 0.3 µN for *β* = 7°) is smaller than the simulated reaction force, which correlates to the smaller thickness of the fabricated springs (see Section 2.3). However, the region with the constant force is also larger, e.g., for *β* = 7°, the CFR starts with a displacement of 97 µm and ends at 267 µm. Figure 9b shows the resulting spring stiffness for exemplary springs that is close to 0 N/m for the constant force range of the springs with a tilting angle of 7° and 8°.

Comparing the experimental results with the simulation presented in Figure 4a, the reaction force is lower, which is attributed to the reduced thickness of the manufactured structures (*t* = 4.8 µm (fabrication) instead of *t* = 5.0 µm (simulation)). This is because the thickness, *t*, of the springs has a cubic influence on the reaction force. The manufactured springs show a lower increase in the reaction force and a smaller influence of the tilting angle on the constant force range. In Figure 4a, the simulated springs with a tilting angle of 4° and 8° do not show a constant force range, while the fabricated springs with these tilting angles show a constant force behavior in Figure 9a.

**Figure 10:**
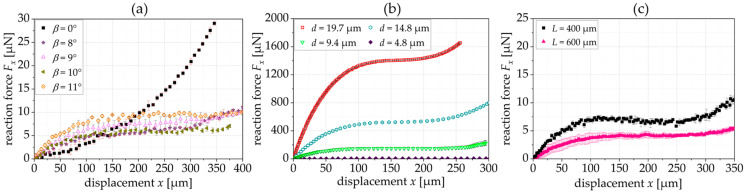
(**a**) Experimentally derived FDC of triangular springs when varying the tilting angle, *β*, (*α* = 30°); (**b**) experimentally shown influence of the spring thickness, *t*, on the reaction force; (**c**) experimentally shown influence of the beam length, *L*, on the reaction force.

As shown in Figure 6c, the inclination angle, *α*, also influences the reaction force. In Figure 10a, we show the corresponding experimental results for a spring with an inclination angle *α* = 30°. To generate a constant force, we need a larger tilting angle when the inclination angle is higher. Figure 10b shows the influence of the spring thickness, *t*, on the reaction force. The reaction force increases with increasing thickness. Thereby, the CFR is becoming smaller. Figure 8c shows that the larger beam segments also achieve larger constant force regions with a slightly smaller reaction force.

## 4. Application in Comb Drive Actuators

An application for the presented springs are comb-drive actuators that struggle with side instability, which means that electrodes guided by the spring escape to the mounted electrodes, limiting the displacement of the comb-drive actuator (cf. Section 2.3). This happens as soon as the first derivate of the electrostatic force with respect to *y* becomes larger than the restoring spring constant in the *y*-direction [1].

**Figure 11:**
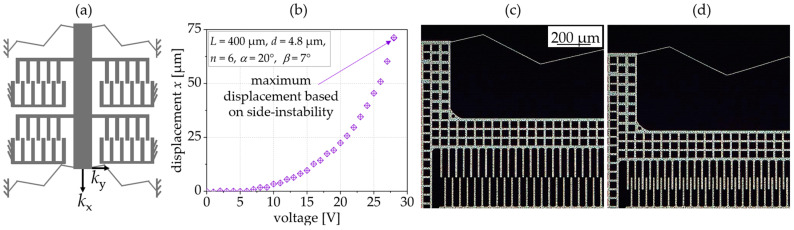
(**a**) sketch of the actuator setup; (**b**) experimentally derived voltage-displacement characteristics of a comb-drive actuator guided by the tilted triangular spring presented in Figure 7 (*β* = 7°); comb-drive actuator at (**c**) 0 V and (**d**) 28 V.

We use the spring characterized in Figure 9 with a tilting angle of *β* = 7° to guide an electrostatic comb-drive actuator. Figure 11a shows a schematic of the setup of the actuator; Figure 11b shows the voltage-dependent displacement with a relatively large displacement at low voltage. The maximum displacement is 71 µm at 28 V, and at larger voltages, side instability occurs. Figure 11c,d show the displacing electrodes.

## 5. Conclusions

In this contribution, we present tilted triangular springs that achieve a partially constant reaction force using a preset tilting angle, *β*. Constant force springs are suitable for mechanisms which control an object with a defined force, as well as for all MEMS in which a mechanical overload could lead to a functional restriction or destruction of the sensitive components. Here, we focus on the influence of the different geometry parameters on the spring reaction force and analyze the spring selectivity based on the tilting angle. An exemplary spring achieves a constant force of 6.8 ± 0.3 µN in the range of 97 µm to 267 µm. We show that tilted triangular springs are very well-suited for guiding large displacement comb-drive actuators due to their high selectivity.

## Figures and Tables

**Table 1:** Parameters of simulated triangular-shaped springs; the varied parameters are in bold.

Analyzed in	*β*	*α*	*n*	*L*	*t*	*d*	Analysis of
Figure 4a–c	**0–8°**	20°	6	400 µm	5 µm	20 µm	reaction force *F_x_*, stiffness *k_x_* and *k_y_*
Figure 5a	**0–8°**	20°	6	400 µm	5 µm	20 µm	selectivity *S*
Figure 5b	**0°–8°**	**10°, 20°, 30°**	**4, 6**	400 µm	5 µm	20 µm	correlation *S*_max._, *L*_proj._ and *β*
**Figure 6a standard spring**	**7°**	**20°**	**6**	**400 µm**	**5 µm**	**20 µm**	**constant force**
Figure 6b	**0–30°**	20°	6	400 µm	5 µm	20 µm	Influence of *β*
Figure 6c	7°	**5–30°**	6	400 µm	5 µm	20 µm	Influence of *α*
Figure 6d	7°	20°	**4–14**	400 µm	5 µm	20 µm	Influence of *n*
Figure 6e	7°	20°	6	**100…500 µm**	5 µm	20 µm	Influence of *L*
Figure 6f	7°	20°	6	400 µm	**3…10 µm**	20 µm	Influence of *t*

**Table 2:** Parameters of selected spring designs.

Analyzed in	*β* [°]	*α* [°]	*n*	*L* [µm]	*t* [µm]	*d* [µm]	*F_x_*_,konst._ (exp.)	*F_konst., range_* (exp.)
Figure 9	0, 2, 4, 6, 7, 8, 10	20	6	400	4.8	20	6.8 ± 0.3 µN (*β* = 7°)	97…267 µm (*β* = 7°)
Figure 10a	0, 8, 9, 10, 11	30	6	400	4.8	20	6.4 ± 1.1 µN (*β* = 10°)	105…355 µm (*β* = 10°)
Figure 10b	7	20	6	400	4.8, 9.4, 14.8, 19.7	20	147.4 ± 1.1 µN (*t* = 9.4 µm)	103…228 µm (*t* = 9.4 µm)
Figure 10c	7	20	6	400, 600	4.8	20	4.1 ± 0.3 µN (*L* = 600 µm)	74…324 µm (*L* = 600 µm)

## Data Availability

The data can be provided by L.S. upon reasonable request.
